# Self-emulsifying drug delivery systems: a novel approach to deliver drugs

**DOI:** 10.1080/10717544.2022.2083724

**Published:** 2022-06-06

**Authors:** Ahmad Salawi

**Affiliations:** Department of Pharmaceutics, College of Pharmacy, Jazan University, Jazan, Saudi Arabia

**Keywords:** Bioavailability, SEDDS, lipophilic, drug delivery, solubility

## Abstract

Self-emulsifying drug delivery systems (SEDDS) are a proven method for poorly soluble substances works by increasing the solubility and bioavailability. SEDDS and isotropic mixtures, are composed of oils, surfactants, and occasionally cosolvents. The ability of these formulations and methods to produce microemulsions or fine oil-in-water (o/w) emulsions after moderate stirring and dilution by water phase along the GI tract might be a promising technique for lipophilic agents with dissolution rate-limited absorption. This review provides an outline of SEDDS's numerous advances and biopharmaceutical elements, types, manufacturing, characterization, limitations, and future prospects. The evaluation of SEDDS and its applications are also discussed, focusing on the advances of SEDDS's solid self-emulsifying delivery mechanism and dosage form. By integrating suitable polymer into the formulation, SEDDS may be studied for the creation of a formulation with sustained drug release. This technology's improvement might lead to a new application in the field of medicine delivery. SEDDS has been demonstrated to be quite efficient in increasing oral bioavailability of lipophilic products. SEDDS is one of the promising methods for controlling the characteristics of medications that are not great choices for oral delivery. It is also worth mentioning that SEDDS may be made in variety of solid dosage forms that are acceptable for both oral and parenteral administration.

## 1. Introduction

Oral delivery of many proteins and medical peptides is limited. Due to the GI tract's enzymatic and absorption membrane limitation, technologies have been investigated to solve these obstacles. SEDDS from the last few years have acquired much interest as prospective carriers for oral peptide and protein administration (Leonaviciute & Bernkop-Schnürch, 2015).

Emulsions serve as drug carriers in pharmaceutical preparations even though they can likely improve the medicine's oral bioavailability by having poor absorption profiles (Zhu et al., 2020). The prominent strategies for enhancing the stability of orally administered APIs are to use delivery systems of drugs that are based on lipids. According to the literature, the terminology for lipid-based techniques is highly debated. The initial droplet size is not the primary factor determining micro and nano emulsions (SMEDDS and SNEDDS). If the droplet size of emulsion is in the nanoscale range, the SNEDDS term should be used. SEDDS are oil and surfactant-based preparations with the help of slow agitation that can be emulsified rapidly in water (Tran & Park, 2021). The chemical structure and physical properties of SEDDS physical qualities were essential determinants of application and tolerance. As a result, these variables must be established at the stage of preformulation (Ujhelyi et al., 2018).

Due to the various possible limitations with the GI system, hydrophilic macromolecular medicines, proteins primarily polysaccharides, therapeutic peptides, and DNA-based therapies, have low oral bioavailability. A range of tactics has been used to address this issue, including structural drug changes, addition of auxiliary agents, and the production of SEDDS nanocarriers, which are used in various studies as a prominent term for both self-nano- and self-micro emulsifying drug delivery systems (SNEDDS/SMEDDS) and emerge to be a successful method for oral medicines (Page & Szepes, 2021). The preparation of SEDDS on an industrial level is economical and simpler than other nanocarriers, including liposomes, micelles, polymer-based nanoparticles, carbon nanotubes, or niosomes, because it is almost like the solution preparation (Mahmood & Bernkop-Schnürch, 2019).

Self-emulsification is influenced by the quality and nature of the concentration of surfactants, pair of oil/surfactant, and oil/surfactant ratio, and the physiological parameters in which it happens, including pH, and temperature. SEDDSs vary from conventional oral drug delivery systems in that digestion of enzymes significantly changes the excipients in the formulation (Amara et al., 2019). Gastric and pancreatic lipases hydrolyze the lipids in the oil phase of SEDDSs in the GIT, releasing additional amphiphilic lipid digestion products. The solubilization of biliary lipids secreted in the bile is quick and these released digested lipids. Different parameters are linked with the gastrointestinal lipolysis process during lipid digestion. These parameters include pancreatic and gastric lipase secretions, the difference in the small intestine’s pH in and the stomach, pH of the lipase action, and secretions of the bile that allow solubilization of micelle by lipolysis products (Park et al., 2020; Sirvi et al., 2022).

SEDDS have also been established to administer hydrophilic macromolecular medications orally like pDHA, peptides, proteins, and polysaccharides throughout the years. The resultant combinations can be integrated into the lipophilic phase of SEDDS because of hydrophobic ion pairing (HIP) with charged auxiliary agents that is lipophilic in nature. By utilizing auxiliary agents in suitable proportions for the HIP, drug release was deliberately modified according to the solubility of the compound in the SEDDS pre-concentrate and the release matrix (de Oliveira & Bruschi, 2022). Based on the target region, the oily droplets might be either mucoadhesive or very mucus permeable. Additionally, coating them with peptides that are cell penetrating, by altering their zeta potential, and their cellular absorption capabilities may be fine-tuned. Meanwhile, several *in vivo* experiments exhibiting bioavailability in the percentage range of single digit have demonstrated SEDDS' potential for oral administration of hydrophilic macromolecular medications. Due to these characteristics, modified SEDDS has shown to be a recent approach of assessment for the administration of hydrophilic macromolecular medications orally (Mahmood & Bernkop-Schnürch, 2019; Cherniakov et al., 2015; Ruiz et al., 2022).

Wide variety of nanocarrier systems are prepared from SEDDS, that appear to be the most appealing, at least from a present industrial standpoint, because their scaling and manufacture are very simple. In a proof of principle investigation, researchers were ready to build the first zeta potential altering SEDDS. But they were able to show that splitting phosphate groups from the surface of SEDDS and altering zeta potential from negative to positive, the shift was rather slight, ranging from −1 to +1 mV in the best scenario (Kang et al., 2021; Tran & Park, 2021). Moreover, excipients like octylamine, cetylpyridinium, or cetrimonium, can be included from a safety standpoint to have positive charges on SEDDs surface that was accessible after the cleavage of phosphate group. Furthermore, because both surfactants (cationic or anionic) were to be included in the same formulation, unwanted ionic interactions, including ion pairing, could not be ruled out (Salimi et al., 2018).

Apart from common methods like dispersibility tests, turbidimetric evaluation, and viscosity tests, complex instrumental requirements like photon correlation spectroscopy (PCS) or dynamic light scattering (DLS), electro kinetic potential measurement, nondestructive spectroscopic techniques (LFDS, FTIR, RS), and numerous microscopic methods (SEM, PLM, EDS) have been defined. To achieve the greatest value, outstanding bioavailability, and tolerance of the dosage forms for human administration, all significant aspects must be identified during the preformulation stage of self-emulsifying drug delivery systems (SEDDS) (Sinka et al., 2022).

## 2. Factor affecting of SEDDS

### 2.1. Dose and nature of drug

Drugs delivered at extremely high doses are not appropriate for SMEDDS unless they show remarkable solubility in at minimum one of the ingredients, particularly the lipophilic phase. SMEDD has the most trouble administering drugs with low water and lipid solubility (usually with log *P* values of about) (Sharma et al., 2012; Akula et al., 2014).

### 2.2. Polarity of the lipophilic phase

The release of drug from microemulsions is regulated through parameters including polarity of the lipid matrix (Rani & Radha, 2021). The HLB, the length of chain and fatty acid degree of unsaturation, and the molecular weight of micronized all impact the polarity of the droplet for their ability to block crystallization and, therefore, establish and sustain the supersaturated state for a longer time (Nigade et al., 2012).

## 3. Benefits of SEDDS as compared to conventional emulsion

Unlike traditional emulsions, which needs high shear to generate a dispersion, SEDDS preparation is simply dissolving the drug in oil and then combining it with surfactants and cosurfactants (Nigade et al., 2012). Creaming, coalescence, breaking, and phase inversion are all common instabilities in traditional emulsions. But on the other side, SEDDS formulations are physically stable because they are isotropic mixtures, clear and are resistant to small temperature changes. Particle size plays an important role as with the increase in particle size solubility of the product increases and the named itself indicates that it has the ability to emulsify when drug delivers at the site of action. This makes better as compared to conventional emulsion which does not emulsify when reaching the targeted site (Wadhwa et al., 2011; Souto et al., 2022). Furthermore, the presentation of final dosage forms of SEDDS formulations can be through hard or soft gelatin capsules of patient compliant. They are compatible with blister or strip packing and guarantees uniformity of dose. For conventional formulations, large containers are required which are unmanageable and there can be a reduction in efficacy as the dispersion media and droplets are non-uniform.

Other benefits of SEDDS include its easy manufacturing by the basic instrument rather than high cost and specialized equipment required by the suspension and emulsion for the monitoring of analytical procedures including rate, intensity, and mixing duration (Betageri, 2019). Transforming liquid-SEDDS into solid dosage forms, which impart physicochemical stability and lower manufacturing expenses while keeping the pharmacokinetic advantages associated with lipids, is a typical strategy used to address these basic shortcomings. For solid-SEDDS development, various approaches of solidification can be used including; *in vivo* emulsification, pre-emulsification, and then *in vitro* stabilization which allows GI tract redispersion of emulsion. Solid-SEDDS may be developed using a variety of solidification techniques, that can be divided into those that (i) emulsify *in vivo* and (ii) are pre-emulsified and stabilized *in vitro*, enabling for emulsion redispersion throughout the GI tract. Solidification may provide a variety of biological benefits to the SEDDS formulation in this way (Joyce et al., 2019). Solidification can provide a variety of biological benefits to the SEDDS formulation including:

### 3.1. Prolonged gastric residence

Polymers like HPMS and microcrystalline cellulose are responsible for the extension of overall transit and gastric emptying time. It causes helpful interactions with epithelial cells of stomach by incorporation of floating excipients which leads to enabling of formulations to be buoyant with the gastric media. This prolongs the total disintegration period as well as the time available for absorption (Setthacheewakul et al., 2011).

### 3.2. Improved intestinal solubility

There are varieties of methods, such as stabilizing supersaturated drug states and regulating digestible lipids’ lipolysis, solidification of SEDDS can increase intestine solubility. Polymeric nanoparticles can be utilized as polymeric precipitator inhibitors (PPIs) for retaining the supersaturated state of solubilized molecules of drug. It also alters the functioning of digestive enzymes by altering in the chemistry and nanostructure of surface of the carrier material. As a result, the precipitation inhibitory action and solubilizing mechanism of lipolysis products increase encapsulated medicinal molecules' intestinal solubility (Joyce et al., 2015).

### 3.3. Improved drug permeability

Mucoadhesive polymers and chitosan, well-known solid-state intestinal permeation boosters are used to manufacture SEDDS to improve medication permeability through the intestinal epithelium. For improvement of permeability, there has been little research on solid-SEDDS. Studies have shown that attachment of silicates with liquid-SEDDS improves intestinal drug permeation, indicating the ability for solid-SEDDS to have potential to deliver class IV drug compounds (Joyce et al., 2019).

### 3.4. Lipid-based oral delivery

According to current parameters, the therapeutic effectiveness of an oral route of administration is increasing. Aqueous solubility, dissolution, and permeability are some of these parameters (Feeney et al., 2016). As per Biopharmaceutical Classification System (BCS), drugs are identified as class II (low solubility, high permeability) or class IV (low solubility, poor permeability). To address all of these challenges, new technologies in the form of innovative dosage forms have been created. It is largely directed at pathogens or diseased cells. Lipid-based formulations increase medication solubilization during GI transit and provide a lipophilic microenvironment to facilitate drug delivery to intestinal absorptive regions (Mohsin et al., 2009). The self-convening ability of lipid has been used to explain several colloidal drug carriers with various structures, including emulsions, micelles, microemulsions, liquid crystalline nanoparticles, vesicular carriers such as solid lipid nanoparticles, liposomes, niosomes, polymer–lipid hybrid nanoparticles, and SEDDS (Rani et al., 2019).

Some natural substances are insoluble in water. For the preparation of systems to transport these compounds, physicochemical approaches and innovations are required. SEDDS have been employed as carriers of hydrophobic chemicals to improve their parameters like solubility and absorption, and bioavailability (de Oliveira & Bruschi, 2022).

### 3.5. Biopharmaceutical issues

It is worth noting that lipids such as triglycerides can influence the oral bioavailability of drug by changing biopharmaceutical features such as improving dissolution rate and enhancing solubility in the intestinal fluid, chemical protection of the drug and enzymatic deterioration in oil droplets, and promoting lymphatic transport of highly lipophilic drugs by forming lipoproteins. The pattern of drug absorption and blood/lymph circulation are influenced by degree of saturation, chain length of, and volume of the lipid.

In many situations, the lipoproteins' lipid core is transported to the circulatory system together with the medicines processed by the intestinal lymph. Lipophilic drugs coupled with lipids have been demonstrated to increase drug absorption into the portal circulation when compared to non-lipid formulations, in addition to boosting lymphatic transport (Caliph et al., 2000).

### 3.6. Specificity

Self-emulsification depends on the ratio of oil/surfactants, its nature of pair, concentration of surfactants, and self-emulsification temperature. Self-emulsifying system (SES), is usually fulfilled through limited and specific combinations of pharmaceutical excipients. The specific physicochemical compatibility of the drug determines the success of the incorporation of the drug into a SEDDS. That is why study of phase diagram and preformulation solubility is needed to prepare suitable formulation design (Tang et al., 2008).

### 3.7. Excipient selection

Self-emulsification is very definite to the nature of the combination of surfactant and oil, the concentration of surfactant and ratio of oil and surfactant as well as the temperature of the occurrence of self-emulsification. The findings support that only extremely precise pharmaceutical excipient combinations led the SESs to be efficient (Shah et al., 1994).

Following the identification of a list of possible excipients, a binary drug–excipient screening for stability, compatibility, and solubility, should be performed to determine the most suited lipid system for the drug in issue. When designing SEDDS/SMEDDS that utilize various excipients, overall solubilizing power of the system should be focused rather than the solubility of the drug in the individual components, even though assessment of affinity and solubility of the drug for each component is essential. Surfactant, co-surfactant, and oil phase components might be synthetic, semi-synthetic, or natural but components are chosen because of (1) attaining maximum loading of drug, (2) to ensure maximum absorption, duration of self-emulsification and droplet size must be kept to a minimum in the gastric environment, (3) to decrease droplet size of emulsion, fluctuation as a function of aqueous medium pH and electrolyte concentration, and (4) to avoid/reduce medication degradation/metabolism in the physiological environment (Rahman et al., 2013; Badadhe & Dalavi, 2022; de Oliveira & Bruschi, 2022).

## 4. Formulation optimization of SEDDS

A comprehensive pharmaceutical approach to formulation creation is indicated as quality by design (QbD) that initiated with predetermined goals and strengthen product and knowledge about the process as well as process control through sound knowledge and assessment of risk. QbD aids in the development of exceptional goods as well as the identification of essential process factors impacting medicinal product production. It also aids in the development of methods for maintaining quality throughout the product's life cycle (Rahman et al., 2013). For the purpose of screening or optimization of the variables, QbD is mostly utilized through design of experiment, which employs different designs such as Plackett–Burman, Box–Behnken, central composite design, mixture design, and fractional factorial design (Betageri, 2019)

### 4.1. Components of SEDDS

#### 4.1.1. Drug

The most important parameter for SEDDS formulation is the lipophilicity and hydrophobicity of a drug. A drug's log *P* should preferably be ≥2. The drug is formulated at a modest dose and should not be subjected to substantial first-pass metabolism (Pouton, 2000).

#### 4.1.2. Oil

Oil is necessary for the lipophilic drugs solubilization. It improves the drug's availability for quick absorption in the GI tract via the intestinal lymphatic system. The degree of esterification and kind of fatty acids and with regard to glycerol to create mono or diglycerides determine the physical, melting, and hydrophilic–lipophilic balance (HLB) features of glycerides. Six to twelve carbon chains present in MCTs and are delivered into the systemic circulation via portal blood. The intestinal lymphatic system transports LCT with more than 12 carbon chains. MCT is the most extensively utilized lipid formulation because of its improved quality of solubility, fluidity, and ability to resist oxidation (Kimura et al., 1994).

By lowering the interfacial tension between the oil and water interface, as well as altering the interfacial film curvature and time, the self-emulsification feature boosts the solubility by minimizing precipitation (Halder et al., 2021).

#### 4.1.3. Surfactant and co-surfactant

Surfactants lower the interfacial tension by forming an interfacial film, allowing for dispersion. During SEDDS formulation, the HLB value must be kept in mind. A surfactant with an HLB value greater than 12 is chosen to achieve better emulsification. It helps to disseminate the intended formulation quickly by forming small oil-in-water (o/w) droplets. Nonionic surfactants are commonly used in the formulation of SEDDS due to their nontoxic nature, despite the fact that they may produce a modest irreversible change in the permeability of the GIT wall. In GIT, a formulation of surface-active compounds that is 30–60% w/w results in improved self-emulsification. Surfactants in high amounts might irritate the wall of the GI tract (Gershanik & Benita, 1996; Matsaridou et al., 2012; Gurram et al., 2015).

**Figure 1.**
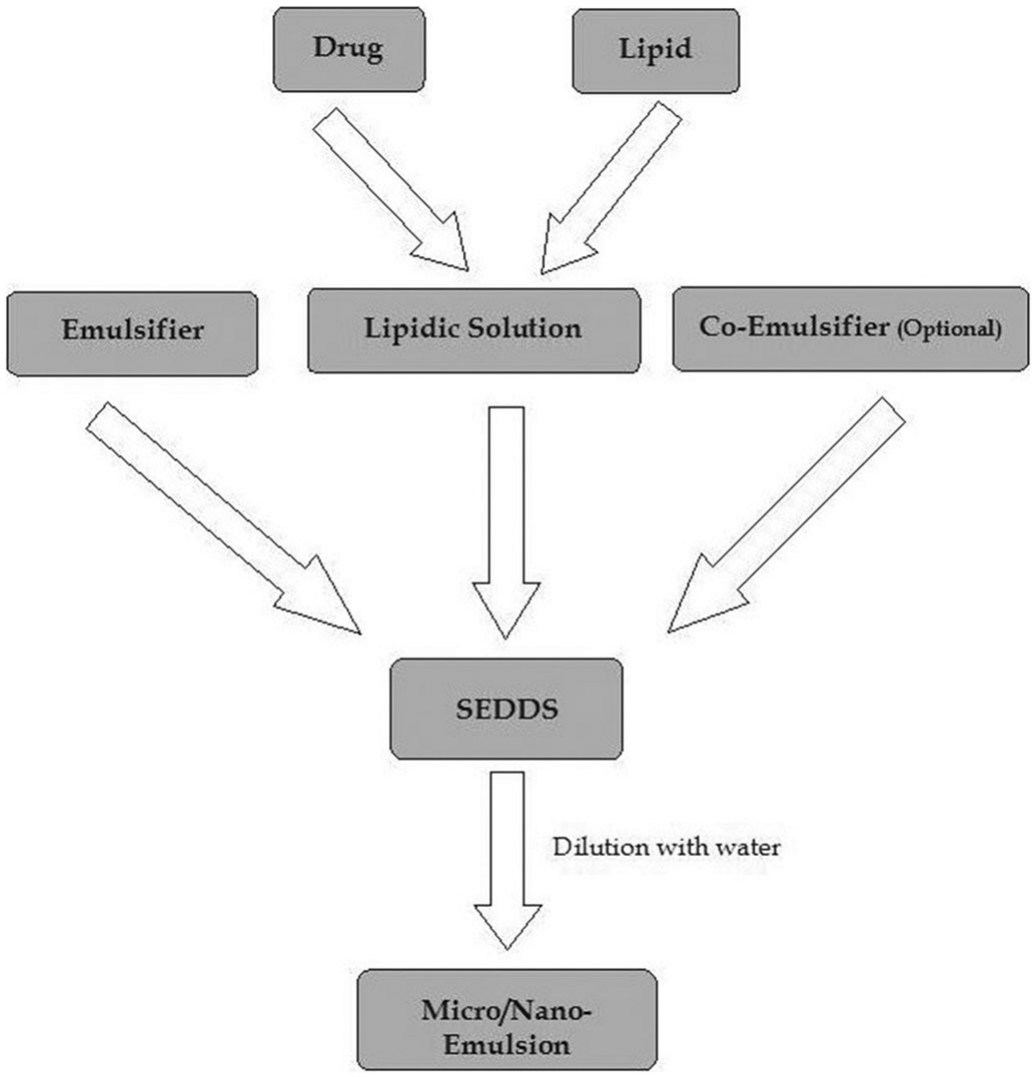
Mechanism of self-emulsifying drug delivery systems (SEDDS).

Co-surfactant lowers the transitory negative value of interfacial tension even further. It gives the interfacial film flexibility so that varied curvatures can be achieved for the creation of different microemulsion concentrations. By adding co-surfactant, the higher amounts of surfactant (approximately 30%) can be simulated (Kohli et al., 2010). The contact enlargement at this moment results in the creation of finely scattered droplets. It will absorb more surfactant or a higher surfactant/co-surfactant ratio until the film is depleted enough to restore positive interfacial tension. Spontaneous emulsion is formed as a result of this. Co-surfactants are typically made up of medium-chain length alcohols (C3–C8) (Rani et al., 2019) (Figure 1).

## 5. Solubility of active drug in SEDDS

The most significant factors for optimizing oral therapeutic effectiveness are pharmaceutical ingredient solubility, dissolution rate, and permeability (Sirvi et al., 2022). SEDDS' dissolution rate is also an essential characteristic since it can alter medication release kinetics and gastrointestinal absorption (de Oliveira & Bruschi, 2022). The water solubility and gastrointestinal permeability are the categories of API as described by the BCS (Amidon et al., 1995). The dissolution of orally administered drugs must be done in the aqueous environment of the gastrointestinal tract, but they must also have a lipophilic feature to pass through the membrane barriers (Cherniakov et al., 2015). The physicochemical compatibility of the medication and the system determines the features of APIs-SEDDS. The oily or surfactant phases are utilized to dissolve the drug of SEDDS. Despite this, the medication can pass through the surfactant interfacial layer (Ruiz et al., 2022). The drug's interaction with the self-emulsification process can also affect API encapsulation efficacy.

One of the simplest procedures for determining the major features of the produced nanoemulsion and its continuous phase is the dye solubilization test (DST). While testing, a water-soluble dye was sprinkled onto the surface of the produced emulsion. The quality of the emulsion's internal and external phases can be established by looking at dye dispersion or clump formation (Ujhelyi et al., 2018).

Poor water-soluble drugs pose a significant formulation challenge because of their high hydrophobicity that restricts them to be dissolved in the solvents medium. Hydrophobic drugs are usually dissolved more effectively by synthetic hydrophilic oils and surfactants than by conventional vegetable oils. The incorporation of solvents such as PG, ethanol, and PEG to the lipid vehicle may also help to increase the solubility of drug (Sareen et al., 2012; Maji et al., 2021).

The success of adding a medicament to a SEDDS is highly reliant on the system's or drug's drug/physicochemical capability. The drug interferes with the self-emulsification process to some extent in the majority of cases, resulting in a change in the optimal surfactant to oil ratio. The efficiency of SEDDS can be changed by preventing charge transport across the system through direct complexation of the drug. The drug molecule is with part of the mixture's components via its interaction with the LC phase, or by penetrating the surfactant interfacial monolayer (Charman et al., 1992).

The impact of the self-emulsification process on the drug may result in a shift in droplet size distribution that varies with the drug's concentration. It is worth noting that in more sophisticated preparations, emulsions with smaller oil droplets are more vulnerable to changes produced by the introduction of therapeutic ingredients. As a result, phase diagram experiments and pre-formulation solubility studies are essential for the development of a suitable SEDDS (Gursoy & Benita, 2004).

## 6. Method of preparation of SEDDS

### 6.1. High pressure homogenizer

High pressure is required for the preparation of nano-formulation. Fine emulsion is formed depending upon the application of high sheer stress. There are two theories that can explain the droplet size including turbulence and cavitation. Nano-emulsion of smaller than 100 nm droplet size can be produced by this method. Various factors are responsible for the production of droplet size of nanoemulsion using high pressure homogenizers, i.e. type of homogenizer, composition of sample and the operating conditions of homogenizer including time, intensity, and temperature. High-pressure homogenization is commonly applied to produce nanoemulsions of food, medicinal, and biotechnological ingredients (Basha et al., 2013).

### 6.2. High energy approach

High mechanical energy is required for the high energy approach which leads the formation of nanoemulsion by mixing surfactants, oil, and co-solvent. Formulation of nanoemulsion extensively uses high energy methods. Strong disruptive forces are provided by the high mechanical energy that are used for breaking up the droplets of large size into droplets of nano size so that nanoemulsions produced would be of high kinetic energy. Basically, SNEDDS require low energy and depend upon the phenomena of self-emulsification (Qian & McClements, 2011).

### 6.3. Micro-fluidization

Micro-fluidizer is a device required by the method of micro-fluidization. The product is pushed toward the interaction chamber by the positive displacement pump. A microchannel is a small droplet channel found in this system. The product formed is then transferred to the impingement area through the microchannels where nanoemulsion of very fine droplets is produced. Then, course emulsion is produced when the mixture of aqueous phase and oil phase is added into the homogenizer. Further processing leads to the formation of a transparent and homogeneously stable nanoemulsion (Patel et al., 2014).

### 6.4. Sonication method

One of the useful methods for the formation of SNEDDS is sonication method. With regard to cleaning and operation, the method of ultrasonication is better as compared to other methods of high energy. In the emulsifications by ultrasonication, the macroemulsions are broken down into nanoemulsion by the cavitation forces provided by the ultrasonic waves. This process reduces the droplet size of the emulsion and leads to an emulsion of nano size. The mechanism of sonication is responsible for the reduction of the droplet size (Mishra et al., 2021).

## 7. Solid SEDDS

A very well-designed system is required for the stabilization of administration of macromolecules through oral route as it is difficult to deliver macromolecules like peptides and proteins. As hydrophilic peptides and proteins tend to precipitate and display structural changes in liquid SEDDS formulations, S-SEDDS has the ability to stabilize them. S-SEDDSs in suppressing first-pass metabolism and P-gp efflux, lymph targeting, and regulated release are all discussed in detail (Tang et al., 2008).

### 7.1. Components of S-SEDDS

Interaction of some definite components including surfactants and oil leads to the process of self-emulsification. For the designing of an efficient system of self-emulsification, three crucial elements are required, i.e. type of surfactant, oil, and the ratios of both oil and surfactant. Low dosage active therapeutic agents with appropriate solubility in lipids, surfactants, and cosurfactants are desirable. Solid SEDDS (S-SEDDS) provide a number of benefits, including regulated drug release, extended gastric residence duration, and enhanced permeability.

Following the development of an emulsion, drugs have a tendency to crystallize and precipitate in the GIT, resulting in an unpredictable pharmacokinetic response. This reduces the amount of medicine that can be loaded into liquid SEDDS, which is a significant drawback when constructing BCS class II and IV pharmaceuticals (Maji et al., 2021).

### 7.2. Solidification of Self-Emulsified formulations

The insulin (Zhang et al., 2012; Sakloetsakun et al., 2013), β-lactamase (Rao et al., 2008), cyclosporine, ritonavir, valproic acid, bexarotene, clofazimine, dronabinol, ibuprofen, and calcitriol (Rajesh et al., 2010; Revathi & Raju, 2012; Bhupinder et al., 2013) were successfully formulated using SEDDS by varying compositions of surfactants and co-surfactants. Some of them are listed in Table 1. 

**Table:** 

Compound	Dosage form	Indication	Reference
Insulin	Capsule	Diabetes	Sakloetsakun et al. (2013)
Cyclosporine	Soft gelatin capsule	Immune suppressant	Bhupinder et al. (2013)
β-Lactamase	Capsule	Enzyme	Rao et al. (2008)
Insulin	Capsule	Diabetes	Zhang et al. (2012)
Ritonavir	Soft gelatin capsule	HIV antiviral	Revathi & Raju (2012)
Bexarotene	Soft gelatin capsule	Antineoplastic	Rajesh et al. (2010)
Ibuprofen	Hard gelatin capsule	Analgesic, antipyretic	Rajesh et al. (2010)
Valproic acid	Soft gelatin capsule	Antiepileptic	Bhupinder et al. (2013)
Fenofibrate	Hard gelatin capsule	Antihyperlipoproteinemic	Revathi & Raju (2012)
Dronabinol	Soft gelatin capsule	Anti-emetic	Revathi & Raju (2012)
Clofazimine	Soft gelatin capsule	Anti-leprosy drug	Rajesh et al. (2010)
Calcitriol	Soft gelatin capsule	Calcium regulator	Bhupinder et al. (2013)

**Table 1.** SEDDS available in the market.

Brand name	API	Company	BCS class	SEDDS use	Dosage form	Ref.
Sandimmun Neoral	Cyclosporin A	Novartis	IV	Immunosuppressant	Soft gelatin capsule	Grevel et al. (1986)
Norvir	Ritonavir	AbbVie	II	To treat HIV/AIDS	Soft gelatin capsule	Singh et al. (2009)
Fortovase	Saquinavir	Roche	IV	To treat or prevent HIV/AIDS	Soft gelatin capsule	Buss et al. (2001)
Agenerase	Amprenavir	GlaxoSmithKline	II	To treat HIV infection	Soft gelatin capsule	Park et al. (2020)
Depakene	Valproic acid	AbbVie	II	To treat epilepsy, bipolar disorder, and seizers	Soft gelatin capsule	Patro & Yadav (2010)
Rocaltrol	Calcitriol	Roche	II	Calcium regulator	Soft gelatin capsule	Mehta & Parekh (2011)
Targretin	Bexarotene	Ligand	II	Antineoplastic agent	Soft gelatin capsule	Lade et al. (2016)
Vesanoid	Tretinoin	Roche	II	For acne	Soft gelatin capsule	Bhattacharya & Prajapati (2015)
Accutane	Isotretinoin	Roche	II	For acne	Soft gelatin capsule	Bhattacharya & Prajapati (2015)

#### 7.2.1. Encapsulation of liquid and semi-solid self-emulsified

This is a simple, common approach for loading low doses of highly potent drugs. The micro spraying and banding procedure are used to fill capsules with liquid self-emulsified solutions. Excipients are heated to 20 °C or above their melting point for encapsulation of semi-solid self-emulsified preparations. Molten mixture is added to load the therapeutic agent. Then, a capsule shell is taken to be filled with drug molten mixture and allowed to cool at room temperature. Banding or micro spray process is used to seal the filled capsule (Lavra et al., 2017).

#### 7.2.2. Spray drying

**Figure 2.**
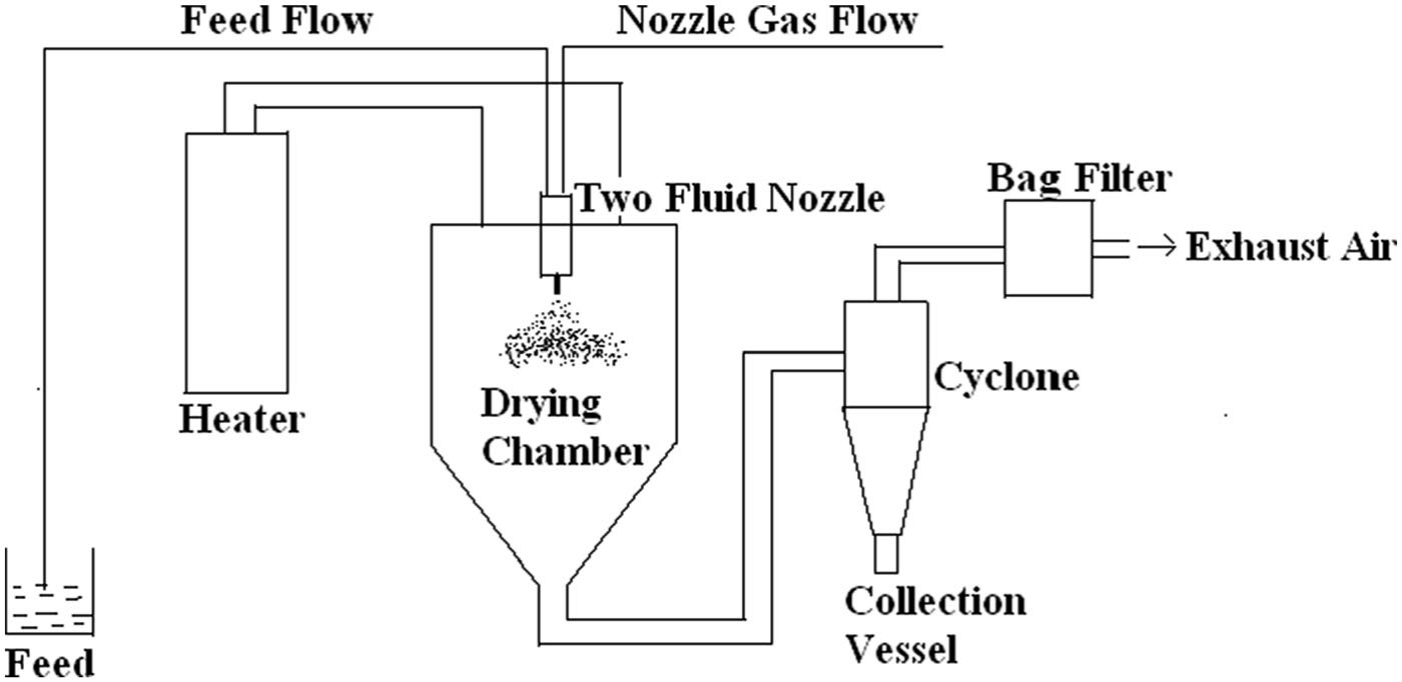
Schematic view of spray drying.

This method involves the mixing of liquid component with solid component by using a solvent as a result of solubilization. After that, the solubilized mixture is atomized into a thin droplet spray. Then a drying chamber is used to dry the fine droplets. The preparation of dried particles is taken place under controlled conditions and flow of air. These particles can then be made into tablet shape. There are a few produced formulations that promote solubility and stability, such as solid dispersion of polyvinyl pyrrolidone (PVP) with nifedipine (20–50%) and anti-HIV efavirenz with soluplus (Figure 2).

#### 7.2.3. Adsorption on to solid carriers

The powders which are free-flowing have greater surface area and the ability to adsorb oil material. The adsorbents are able to adsorb 70% of SEDDS in liquid form. By simply combining the mixture, the liquid self-emulsifying compositions are adsorbed onto this free-flowing powder.

#### 7.2.4. Melt granulation

The use of a binder is used in this procedure to create powder agglomeration. At relatively low temperatures, the binders are melted or softened. It has significant advantages over traditional wet granulation because it is single step process that eliminates the incorporation of liquid components and subsequent drying phases. Few factors to be managed while processing are mixing time, impeller speed, particle size, and binder viscosity (Hauss et al., 1998).

#### 7.2.5. Spherization on extrusion/melt extrusion

Extrusion aid is introduced into the liquid self-emulsifying formulation first. After that, the mixture is mixed with water. It extrudes from a die using force while maintaining constant temperature, pressure, and product flow (Rani et al., 2019).

## 8. Super-saturable SEDDS and pharmacokinetic parameters

The solubility of the compounds can be maintained above its equilibrium state by the phenomena of supersaturation and without the precipitation resulting in the crossing of biological membrane by various molecules of drugs. Due to inadequate solubility, about 70% of prospective drug molecules failed to attain the greatest degree of therapeutic action. It causes a slower rate of absorption. There are a variety of alternative approaches, such as cyclodextrin complexation, solid dispersion, and lipid-based drugs, but SEDDS-based formulation has received a lot of attention for its ability to overcome the solubility issue.

SEDDS-based formulations have been described for a variety of drug types, including anti-cancer, anti-viral, antidiabetic, natural, and antibiotic products. A supersaturated (S) solution has a high concentration of drug, which can increase the driving power for flux across the GI membrane, resulting in increased absorption for a long period of time (Kataoka et al., 2012).

By delaying nucleation and crystal development in the aqueous media, polymers utilized in the manufacture of S-SEDDS formulations kinetically and thermodynamically impede drug molecule precipitation. For poorly water-soluble pharmaceuticals, a variety of SSEDDS formulations, like silybin and celecoxib, were investigated, and it became obvious that the polymers employed in these formulations inhibit precipitation, making them a superior alternative for improving oral bioavailability and absorption.

Polyvinyl pyrrolidone polymer has extensively been used for the improvement of solubility of water of many drugs of poor solubility like estradiol, sulfathiazole, chlorothiazide, fluocinonide, phenytoin, norethindrone acetate, and hydrocortisone acetate (Rani et al., 2019).

### 8.1. Mechanism of SEDDS

A quick study of the literature indicates a variety of microemulsion generation methods. The generation of microemulsion droplets is thought to be caused by surfactant-mediated intricate film formation at the oil–water interface. Emulsification happens when the transformation in entropy favoring dispersion is better than the energy required for dispersion surface area amplification and the free energy (*G*) is negative, according to the thermodynamic theory of microemulsion production (Tenjarla, 1999). The energy necessary to establish a new surface between the two phases is connected to the free energy in the microemulsion production, as shown in the equation below:

ΔG=ΣNr2σ

where Δ*G* represents the process's free energy, *N* is the number of droplets, *r* is the radius, and *σ* is the interfacial energy. The two emulsion phases will most likely split, reducing the interfacial area and therefore the system's free energy. Surfactants stabilize the emulsion that arises from aqueous dilution by establishing a single layer around the emulsion droplets, lowering interfacial energy, and preventing coalescence (Rajpoot et al., 2019).

## 9. Dosage form of SEDDS

### 9.1. Self-emulsifying capsule

When capsules carrying liquid solution SE preparations are delivered, microemulsion droplets form in the GIT and disperse to reach the area of absorption. If the microemulsion's phase separation is permanent, no increase in medication absorption may be expected. To solve this problem, sodium dodecyl sulfate was added to the SE formulation.

### 9.2. Self-emulsifying sustained/controlled release

The use of surfactants and lipids in the preparation of SE tablets has shown tremendous promise. SE pills are really helpful in preventing negative effects. Incorporating indomethacin into SE tablets, for example, may improve the drug's penetration across the GI mucosal membrane, thus lowering GI bleeding.

### 9.3. Controlled/sustained release self-emulsifying pellets

Pellets can provide variety of advantages over conventional solid dosage forms, including production flexibility, lower intra- and inter-subject fluctuation in plasma profiles, and less GI discomfort without compromising drug absorption. Solid dispersions that self-emulsify: solid dispersions may increase the dissolving rate and bioavailability of drugs that are water insoluble, but they still have manufacturing and stability concerns. Using SE excipients can help you overcome these obstacles (Nigade et al., 2012).

### 9.4. Semisolid SEDDS

Semisolid SEDDS are synthesized *in situ* utilizing lipidic ingredients comparable to those used in liquid SEDDS, but with a higher melting point than room temperature. These formulations are unique in that they do not contain cosurfactants, but only comprise lipids and surfactants. For the manufacturing of semisolid SEDDS lauryl macrogel-glycerides including gelucire 44/14, gelucire 50/13, derivatives of polyoxyethylene hydrogenated castor oil including cetyl alcohol derivative, Nikkol HCO50, and polyoxyethylene polyoxypropylene block polymer are the most frequently used surfactants and lipids. Such preparations have a greater viscosity than the comparable liquid SEDDS, resulting in increased medication stability and mobility during handling.

However, because of lipids with high melting point, these formulations generally show poor emulsification efficiency *in vivo*, likely contributing to uneven drug absorption patterns. Several cases on the semisolid SEDDS, such as carvedilol 93 and atorvastatin 94, have been for increasing their oral bioavailability. It was also revealed that semisolid SE formulations produced with glyceryl mono/dicaprylate, diethylene glycol monoethyl ether, propylene glycol monocaprylate, and gelucire 44/14, have improved physicochemical characteristics, due to the supersaturation which prevents drug precipitation, these formulations showed significant levels of resistance to dilution and stability (Singh et al., 2014).

### 9.5. Self-emulsifying hybrid microparticles

These are colloidal solid SE systems made up of a mix of medium chain triglycerides and silica microparticles with particle sizes ranging from 3 to 100 nm. The liquid oily formulations are encapsulated in microparticles in this method, which may then be given in hard gelatin capsules or compacted into tablets. Spray drying of lipidic emulsions having positively charged lipophilic surfactants and colloidal silica particles in aqueous phase produces microparticles. Under *in vivo* circumstances, high drug loading, higher drug absorption due to the presence of cationic charge on the surface, and improved drug stability are all benefits of such systems.

Drugs like celecoxib, telmisartan, and theophylline for which silica-based lipidic SE microparticles have been tested to see whether they might improve bioavailability. In beagle dogs, formulations of SE lipid-hybrid microparticles of celecoxib containing Capmul MCM and Aerosil 380 showed a more than twofold increase in fed state oral bioavailability and a 6.5-fold increase in fasted state oral bioavailability when compared to a conventional lipidic solution, as well as a significant reduction in arthritis-like conditions.

The bioavailability of telmisartan is found to be increased by 154% formed as mesoporous silica nanoparticles, as well as greater cellular absorption and minimal toxicity in Caco-2 cell lines, as compared to the drug's traditional formulation. When compared to a traditional emulsion, silica–lipid hybrid microcapsules loaded with celecoxib showed a nearly twofold increase in *C*_max_ and a 93% improvement in relative oral bioavailability (Tan et al., 2009).

### 9.6. Self-emulsifying nanoparticles

Oily liquid compositions are enclosed in SE nanoparticles. Such formulations are created utilizing a solvent injection process and an appropriate blend of polymers like polylactic acid (PLA), polyglycolic acid (PGA), and polyglycolic acid-co-glycolic acid (PLGA). The nanoparticles give a regulated drug delivery profile, better stomach fluid stability, and increased oral bioavailability. When these formulations come into touch with GI fluids, they create o/w microemulsions *in situ*. 5-Fluorouracil and paclitaxel are two medicines that have recently been suggested to be made into SE nanoparticulate systems in order to investigate their oral bioavailability increase.

As evidenced by MTT testing, TUNNEL method, and immunohistochemical staining, the SE nanoparticles of 5-FU using PLGA/O-carboxymethyl chitosan showed dramatically improved cellular absorption of drug through the intestinal lymphatic routes, decreased cytotoxicity, and noteworthy reduction in gliomas. With the help of chitosan and glyceryl monooleate as emulsion solvent evaporation, SE nanoparticles of paclitaxel were shown to have a fourfold increase in cellular absorption and much decreased cytotoxicity in the MTT experiment (Trickler et al., 2008).

### 9.7. Self-emulsifying controlled release tablets

Self-emulsifying controlled release tablet (SECRET) is a more recent technical advancement in the S-SEDDS field for producing a controlled drug release profile. SECRET is a patented proprietary platform technology created by AlphaRx Inc. (Markham, Canada), in which tablets are formed with the help of liquid SE formulations by adsorbing onto the surface of rate-controlling polymers like HPMC, HPC, and others. These aid in the long-term release of the medication from the polymer matrix. Including systems have important meritorious features in formulation creation, such as site-specific delivery and improved intestinal wall permeability and solubility to aid medication dispersion in the gastrointestinal tract.

The coenzyme Q10 SE controlled release hydrophilic matrices which use Avicel-112 and Kollidon V64 as release-controlling polymers, have improved drug stability and controlled release properties significantly. The composition of SE tablets of carvedilol includes Aeroperl, MCC, HPMC, that significantly increase *in vitro* drug absorption in HCT-116 cell lines, perhaps owing to P-gp efflux inhibition.

The capacity of solid SMEDDS tablets containing candesartan cilexetil dramatically increases the pace and extent of drug dissolution, that proves the better oral bioavailability. Diclofenac SE pellets produced with natural ingredients like goat fat and Tween 80 similarly showed a prolonged release profile of drug release.

### 9.8. Self-emulsifying controlled release capsules

These are made by coating liquid-filled soft-gelatin capsules with a thin layer of semipermeable polymeric material. The lipidic SE formulations give the necessary therapeutic activity for a prolonged length of time due to their semipermeable character. An inflatable layer can also be added to the semipermeable layer to adjust the rate of medication release from the capsule shell. Cardiovascular medications, antiretrovirals, anticancer treatments, corticosteroids, and immune suppressants such as nimodipine, cyclosporine, ritonavir, dexamethasone, vinblastine, and mepitiostane have all been reported to benefit from this method.

The semipermeable coating is made up of cellulose acetate, cellulose acetaldehyde dimethyl acetate, cellulose acylate, and polyurethanes, while the expandable coat on the gelatin shell is made up of Carbopol, sodium carboxymethyl cellulose (CMC), hydroxyethyl cellulose (HEC), HPC, HPMC, and other materials. By converting SE formulations into S-SEDDS using solidifying adsorption carriers, efficient delivery of SE formulations may be achieved. For controlled drug release over long periods of time, such formulations are encapsulated in mini capsules coated with sustained release polymers such as PVP K30 and acrylic resins (Singh et al., 2014).

## 10. Characterization of SEDDS

### 10.1. Visual evaluation

Visual observation helps in the assessment of self-emulsification. The existence of a clear, isotropic, transparent solution after water dilution of SEDDS suggests microemulsion production, whereas an opaque, milky white appearance indicates macroemulsion evolution. A lack of precipitation and/or phase separation suggests that the formulation is stable.

### 10.2. Analysis of droplet size

The size of the droplet is determined by the surfactant's type and concentration. The microemulsion generated during dilution of SMEDDS with water has a very narrow droplet size distribution, which is critical for optimal drug release, *in vivo* absorption, and stability. Droplet size analysis is done using DLS methods.

### 10.3. Zeta potential measurement

The zeta potential reflects the emulsion's stability following dilution. If the zeta potential is larger, the formulation remains stable. When compared to particles with either surface charge, particles with a zwitterion charge exhibit greater biocompatibility and a longer blood residence period (Balakrishnan et al., 2009).

### 10.4. Emulsification time

The amount of time it takes to emulsify a formulation is determined by the oil/surfactant and oil phase ratio. This is determined using a basket dissolution equipment, which observes the development of a clear solution under agitation following drop wise formulation addition to a water-filled basket (Elnaggar et al., 2009).

### 10.5. Cloud point determination

The cloud point of a homogeneous solution is the temperature at which it drops its transparency. Above the cloud point, the surfactant normally loses its ability to form micelles. It is determined by progressively raising the temperature of the formulation and spectrophotometrically detecting the turbidity. The cloud point of the surfactant is the temperature at which the percentage transmittance decreases. To maintain self-emulsification, formulations should have a cloud point higher than 37.5 °C (Elnaggar et al., 2009).

### 10.6. Viscosity measurements

A rheometer, Brookfield viscometer having a cone and plate with rotating spindle is used to assess the viscosity of diluted SMEDDS formulations that are microemulsions (Betageri, 2019).

### 10.7. Liquefaction time

This analysis is performed to determine how long it takes for S-SEDDS to melt in a simulated GI environment without moving. The dosage form, which is threaded to the bulb of a thermometer, is covered in a transparent polyethylene film. The thermometer should then be placed in a round bottom flask with 250 mL of simulated stomach juice without pepsin and held at 37 °C. After that, the time it takes for the liquefaction to happen is noted.

### 10.8. Nuclear magnetic resonance (NMR) studies

These methods are utilized to investigate the dynamics and structure of microemulsions. Self-diffusion assessments utilizing several tracer approaches, most often radio labeling, provide information on the components' mobility and microenvironment. The magnetic gradient on the samples is used in the Fourier transform pulsed-gradient spin-echo (FT-PGSE) methods, which enables for the simultaneous and quick measurement of the self-diffusion coefficients of several components. The Stokes–Einstein equation may be used to compute the self-diffusion coefficient.

D=KT/6πηr

where *T* is the absolute temperature, *η* is the viscosity, *K* is the Boltzmann constant, and *r* is the radius of droplet.

### 10.9. Scattering techniques

For the investigation of microemulsion, scattering approaches have been used. Small-angle X-ray scattering (SAXS), DLS, PCS, and small angle neutron scattering (SANS) are some of the techniques used. Structural data provided by SAXS on macromolecules vary in size from 5 to 25 nm, as well as repetition distances in partly ordered systems up to 150 nm in partially ordered systems. It is used to determine the structure of particle systems at nanoscale or at microscale, including size of particles, dispersion, morphologies, and the surface-to-volume ratio, among other things. To use SANA is to find droplet shape and size. Micelles, oil-swollen micelles, and mixed micelles, are described by the term 'droplet'. The interference effect of wavelets dispersed from diverse materials in a sample is used in small-angle neutron scattering investigations.

The dilution of the sample necessary to reduce interparticle interactions is a fundamental disadvantage of these approaches. The structure and content of the pseudo ternary phases can be altered by this dilution. Despite this, effective determination has been achieved utilizing a dilution procedure that preserves the droplet identity. Incorporating deuterated molecules or protonated, SANS allows for selective increase of the scattering ability of distinct microemulsion pseudo phases. The variation in the frequency of the scattering by the droplets due to Brownian motion is studied using DLS and PCS (Rahman et al., 2013).

### 10.10. Test of thermodynamic stability

Physical stability is essential for a formulation's performance, as precipitation of the chemical in the excipient matrix might have a detrimental influence. Excipient step separation can occur as a result of inadequate formulation physical stability, lowering bioavailability, and decreasing therapeutic effectiveness. Brittleness, softness, and delayed or partial drug release may arise from incompatibilities among the formulation and the gelatin shell of the capsule. The following cycles are used to carry out these investigations.

### 10.11. Turbidimetric test

Turbidity is a measurable characteristic that may be used to estimate droplet size and self-emulsification time. After a given amount of SEDDS is administered to a fixed amount of suitable medium under continual stirring at 50 rpm on a magnetic stirrer at optimal temperature, the turbidity is measured using a turbidity meter. As the time required for complete emulsification is too short, the rate of turbidity shift, or rate of emulsification, cannot be measured. Turbidimetric analysis is used to track the growth of droplets following emulsification (Betageri, 2019).

### 10.12. Determination of self-emulsification time

Using a primitive nephelometer and a rotating paddle to assist emulsification, we investigated the efficiency of emulsification of several formulations of Tween 85/medium-chain triglyceride systems. This allowed the emulsification period to be measured. Samples were obtained for particle size using photon similarity spectroscopy after emulsification, and self-emulsified and homogenized systems were compared. The self-emulsification process was studied using light microscopy. The process of emulsification was precisely defined as the erosion of a thin cloud of microscopic particles off the surface of big droplets, rather than a steady decrease in droplet scale (Halim et al., 2021).

## 11. Limitations

The absence of reliable predictive *in vitro* models for the assessment of SEDDSs and other lipid-based formulations is one of the barriers to their development. Traditional dissolution procedures are ineffective because these formulations may be dependent on gut digestion prior to drug release (Chen et al., 2010). An *in vitro* model of the duodenum's digestion processes has been constructed to imitate this. Before the strength of this *in vitro* model can be assessed, it must be refined and validated. In addition, because development will be based on *in vitro*–*in vivo* correlations, several prototype lipid-based formulations must be produced and evaluated *in vivo* in an appropriate animal model. Chemical instability of medications and high surfactant concentrations in formulations (about 30–60%) that irritate the GIT are a few other downsides. Furthermore, it is known that volatile cosolvents in traditional self-micro emulsifying formulations diffuse into the shells of soft or hard gelatin capsules, causing lipophilic drugs to precipitate. Due to the dilution impact of the hydrophilic solvent, the drug's precipitation propensity may be increased when diluted. Simultaneously, validating formulations with several components becomes more difficult (Rajpoot et al., 2019).

## 12. Applications

Lipids, surfactants, and cosolvents make up the SEDDS formulation. The system may form an o/w emulsion when separated by a water phase with modest stirring. SEDDSs deliver medications in small droplets with a balanced distribution, resulting in improved dissolution and permeability. As medicines can be loaded in the inner phase and supplied via lymphatic bypass sharing, SEDDSs protect drugs from enzymatic hydrolysis by in the GI tract and decrease presystemic clearance in the GI mucosa and hepatic first pass metabolism (Kumar et al., 2010).

## 13. Future perspectives

In general, the technique appears to be highly advanced to efficiently control the enzymatic, sulfhydryl, and mucus barriers, giving convincing benefits over presumably all other delivery strategies in this area. SEDDS, on the other hand, has yet to attain its full potential in terms of overcoming the gut epithelial barrier. Improved information and comprehension of the destiny of HIPs and SEDDS on the intestinal epithelium is undoubtedly a precondition for such advancements.

The first adjusting confocal/STED-laser microscopic evaluations of the cell uptake actions of peptides, surfactants, HIPs, and SEDDS marked with various colorful fluorescence dyes disclosed a wide range of achievable connections with epithelial cells, such as deposition of droplet on the cell membrane, SEDDS fusion with the cell membrane, and indeed the utilization of intact HIPs and droplets into cells. The majority of the parameters that govern HIPs and SEDDS' fate on the intestinal epithelium are unknown, but they are critical for building more effective SEDDS for oral macromolecular drug delivery. For example, the stability of HIPs in the intestinal fluid and on the cell, membrane appears to be one of them. The zeta potential and the kind of surfactants employed in SEDDS, both of which have permeation-enhancing qualities, are likely to be important considerations (Mahmood & Bernkop-Schnürch, 2019).

SEDDS' medicinal and economic potential has been greatly enhanced by the discovery of solid-SEDDS. Solid-SEDDS are considered state-of-the-art delivery vehicles for poorly water-soluble pharmaceuticals because to improve loading of drug, stability, precision dose, ease of processing and storage, and higher patient satisfaction. While developing solid-SEDDS has gotten a lot of interest, there has not been much study done on the essential formulation characteristics that affect *in vivo* drug absorption. Similarly, only a few studies have compared the delivery performance of solid-SEDDS to that of liquid-SEDDS predecessors (Schirm et al., 2003).

The extensive systematic research of the molecular relation between drug molecules, solid carriers, and lipid excipients are required to maximize *in vivo* absorption of drug molecules encapsulated in solid-SEDDS and unveil their full therapeutic value. Compare and contrast the solubilization behavior and *in vivo* pharmacokinetics of numerous drugs encapsulated in liquid- and solid-SEDDS to begin. Second, in order to explore and test the relationships within solid-SEDDS on the nanoscale, it is proposed that a variety of impactful physicochemical, biophysical analysis techniques, and surface sensitive, be used for the clarification of the optimal parameters that ultimately leads to the improvement of biopharmaceutical performance for given therapeutics (Joyce et al., 2019).

## 14. Conclusions

SEDDS are a viable formulation method for medicinal molecules with low water solubility. SEDDS have been demonstrated to remarkably enhance oral bioavailability, and utilized to orally administer hydrophobic medicines. The efficacy of the SEDDS in many circumstances, formulation is case specific; hence, the composition of the SEDDS formulation must be thoroughly assessed. As SEDDS preparations often use very high amounts of surfactants, the surfactant’s toxicity should be noted. In reality, a balance must be struck between the surfactant's toxicity and its propensity to self-emulsify before it can be used. Two additional critical parameters that impact GI absorption effectiveness include charge and size of the oil droplet in the emulsion produced. As an alternative to standard SEDDS, numerous preparations have been created to provide modified emulsified formulations. Self-microemulsion formulations, preformulated freeze-dried emulsions, surfactant dispersions (Tsuji et al., 1996), microencapsulated emulsions, pellets that are self-emulsifiable, solid SESs and lipid/crosslinked polymeric matrices are just a few examples. Upon water dilution, all of such formulations will yield fine oil droplets or micelle dispersions. Currently, pharmacological products developed as SEDDS, such as CsA, ritonavir, and saquinavir, are freely accessible on the market. As roughly 40% of novel drug compounds are hydrophobic, it predicts that further drug products for the pharmaceutical industry will be formed as SEDDS in the coming years (Gursoy & Benita, 2004).
